# Classification of sleep apnea based on EEG sub-band signal characteristics

**DOI:** 10.1038/s41598-021-85138-0

**Published:** 2021-03-12

**Authors:** Xiaoyun Zhao, Xiaohong Wang, Tianshun Yang, Siyu Ji, Huiquan Wang, Jinhai Wang, Yao Wang, Qi Wu

**Affiliations:** 1 Chest Clinical College, Tianjin Medical University, Tianjin, 300222 China; 2 School of Life Sciences, Tiangong University, Tianjin, 300387 China; 3 Respiratory and Critical Care Medicine Department and Sleep Center, Tianjin Chest Hospital, Tianjin, 300222 China; 4 School of Electronics and Information Engineering, Tiangong University, Tianjin, 300387 China; 5 School of Precision Instruments and Optoelectronics Engineering, Tianjin University, Tianjin, 300072 China; 6 Department of Respiratory and Critical Care Medicine, Tianjin Medical University General Hospital, Tianjin, 300052 China

**Keywords:** Data processing, Machine learning

## Abstract

Sleep apnea syndrome (SAS) is a disorder in which respiratory airflow frequently stops during sleep. Alterations in electroencephalogram (EEG) signal are one of the physiological changes that occur during apnea, and can be used to diagnose and monitor sleep apnea events. Herein, we proposed a method to automatically distinguish sleep apnea events using characteristics of EEG signals in order to categorize obstructive sleep apnea (OSA) events, central sleep apnea (CSA) events and normal breathing events. Through the use of an Infinite Impulse Response Butterworth Band pass filter, we divided the EEG signals of C3-A2 and C4-A1 into five sub-bands. Next, we extracted sample entropy and variance of each sub-band. The neighbor composition analysis (NCA) method was utilized for feature selection, and the results are used as input coefficients for classification using random forest, K-nearest neighbor, and support vector machine classifiers. After a 10-fold cross-validation, we found that the average accuracy rate was 88.99%. Specifically, the accuracy of each category, including OSA, CSA and normal breathing were 80.43%, 84.85%, and 95.24%, respectively. The proposed method has great potential in the automatic classification of patients' respiratory events during clinical examinations, and provides a novel idea for the development of an automatic classification system for sleep apnea and normal events without the need for expert intervention.

## Introduction

Sleep apnea, a common sleep disorder, is characterized by the frequency of more than five apneas per hour during sleep or the stopping time of respiratory airflow for more than 10 s^1,2^. Sleep apnea can cause daytime fatigue, a lack of energy, impaired memory, and may also cause several diseases, including high blood pressure, coronary heart disease and stroke^3^. There are three types of sleep apnea, including obstructive sleep apnea (OSA), central sleep apnea (CSA), and mixed apnea (MSA)^4^. Among these, OSA accounts for the largest proportion. It manifests as repeated upper respiratory tract attacks, where the respiratory rhythm is still maintained. In CSA, respiration is either diminished or is completely absent. MSA is a mixture of these two types, defined as a central apnea in a relatively short time interval followed by obstructive apnea^4^. When conducting a diagnosis, the type of sleep apnea that each patient has is determined by the specific type of sleep apnea event. Patients with OSA mostly experience events obstructive events, while patients with CSA mostly experience central events. Mixed events are usually accompanied by both central and obstructive events. In the event of mixed events, if obstructive event is dominant, it is diagnosed as OSA. Otherwise, it is diagnosed as CSA^5^. Sleep apnea hypopnea index (AHI) refers to the number of apneas plus hypopneas per hour of sleep, and is related to the severity of apneas. According to Chicago standards, the severity of apnea is classified into four categories, including normal (AHI<5), mild sleep apnea syndrome (5<AHI<15), moderate sleep apnea syndrome (15<AHI<30), and severe sleep apnea syndrome (AHI>30)^6^.

The gold standard for conducting a diagnosis of sleep apnea is through the use of sleep polysomnography^7^, which is a set of variables that record a variety of electrophysiological and pneumological signals continuously and simultaneously^8^. The signals that are recorded include electroencephalogram (EEG), electrocardiogram (ECG), electromyogram (EMG), electro-oculogram (EOG), oronasal airflow, ribcage movements, abdomen movements and oxygen saturation^9^. However, this method of diagnosis requires sleep technologists to monitor and diagnose sleep apnea events, which is complicated, expensive, and time-consuming^10^. Therefore, there is a need for an automatic method of diagnosis for sleep apnea events that are both low cost and can be carried out in a relatively simple manner.

In recent years, an increasing number of studies have used the characteristics of a single signal to carry out an automated diagnosis of sleep apnea events^11^. Using respiratory airflow, oxygen saturation (SpO_2_), and photoplethysmography (PPG) alone can help identify apnea events, which have achieved many research results^12,13^. Although these signals have the advantage of being easily acquired, they have certain limitations. First, the diagnosis of sleep apnea is based on sleep staging^14^. These methods cannot judge a patient's sleep state and sleep stage, while may cause deviations with regards to diagnosis of sleep apnea. EEG signals are commonly used in sleep staging, as the use of EEG signals can help ensure the patient's sleep state, and the classification of sleep apnea events is relatively accurate. Secondly, in certain scenarios, such as during any chest and abdomen surgeries or limb surgeries, there is no or inconvenient respiratory airflow monitoring, SpO_2_ monitoring, and PPG monitoring. Even for patients that suffer from irregular breathing or obvious arrhythmia, respiratory airflow monitoring, SpO_2_ monitoring, and PPG monitoring may lose their utility. However, the use of EEG signals alone in the above cases can help identify sleep states, as well as various apnea events, which have important clinical significance. In addition, EEG signals reflect electrical activity of the brain and can obviously be used to evaluate sleep stages and problems^15–17^. At the same time, sleep quality is affected by sleep problems such as sleep apnea events^18^. In summary, the main purpose of this research is to classify sleep apnea events based on EEG signals.

Many studies emphasize the function of EEG signals in the automatic classification and detection of sleep apnea^19,20^. Talluk et al. used bispectrum analysis to extract the secondary phase coupling amount of each frequency band as a feature of EEG^21^. Almuhammadi et al. used the energy and variance of brain electronic band signals as characteristics that would allow identification of OSA patients from normal control groups, and achieved an accuracy of 97.14%^22^. Zhou et al. used trend fluctuation analysis (DFA) to extract the scale index of EEG signals as features, and used support vector machine (SVM) to identify apnea patients, which led to an average accuracy of 95.1%^23^. These studies are able to distinguish sleep apnea patients from healthy subjects, but the automatic detection of sleep apnea patients' apnea events is an important task. There has been abundant literature reporting use of EEG signals to study the automatic classification of sleep apnea events. What these studies have in common is that they are only able to distinguish between sleep apnea and non-apnea events, but do not involve the specific subclassification of apnea event types (OSA or CSA). Bhattacharjee et al. used the Rice density function to model the feature changes of sub-frames, and used these modeling parameters and other statistical parameters as the input of the K-nearest neighbor (KNN) classifier, which led to an accuracy rate of 98.02%^24^. Saha et al. proposed feature extraction using multi-band entropy methods, and reported 87.64% as the average accuracy of the KNN classifier. They also characterized the energy ratio between frequency bands of multi-band EEG signals, which led to an average accuracy of 92.21%^25^. Ahmed et al. obtained statistical features from the time pattern of beta band energy and applied them to the classifier, leading to an average accuracy of 82.28%^26^. Shahnaz et al. adopted the feature extraction method based on the power ratio of incremental frequency band of the Delta sub-frame. When using SVM and KNN, the average accuracy was found to be 84.07% and 84.83%, respectively^27^. Sachin et al. proposed a Hermite decomposition algorithm based on particle swarm optimization (PSO), which utilized the Hermite function (HFs) optimized by particle swarm optimization to extract Hermite coefficients from EEG signals to identify SA events. The classification accuracy rate was 98.82%^28^. Additionally, the Hermite function (HFs) optimized by the evolution technology (ETS) was utilized to represent the EEG signal, and the artificial bee colony (ABC) algorithm was carried out for feature extraction based on Hermite coefficients, which led to an accuracy as high as 99.53%^29^.

Correctly detecting and distinguishing apnea events is the first step in diagnosing the type of apnea. The main purpose of this study is to develop a method for automatic classification of OSA events, CSA events and normal breathing events, based on feature extraction of EEG sub-band signals. After preprocessing the data, the features of the EEG signal were obtained, and the set after feature selection was used as the input for the classifiers. Finally, the effectiveness of the method was evaluated.

## Materials and methods

### EEG data

The data used in this study was obtained from the night polysomnography monitoring database of patients in the Tianjin Chest Hospital. The data is composed of overnight polysomnography data recorded by the PHILIPS RESPIRONICS ALICE5 sleep monitor, that was made in the United States. The C3-A2 and C4-A1 EEG signals of 30 patients were selected on random as the research subjects. The 30 patients included 23 men and 7 women, between the ages of 37-78 (average 55.17 ± 11.90 years), with a BMI range of 19.83–39.26 kg/m^2^ (average 29.20 ± 4.47 kg/m^2^), and an AHI range of 8.2-68.9(average 29.18 ± 4.46 events/h). All normal breathing events or apnea events were taken during the patient's sleep and were marked by experienced sleep experts according to the AASM 2012 guidelines. Among them, from the overnight sleep data of 30 patients, there were 1229 epochs of OSA events, 812 epochs of CSA events, and 1418 epochs of normal breathing events. The sampling frequency of the EEG signal was 100 samples/second.

### Ethics approval and consent to participate

This study was approved by the Ethics Committee of Tianjin Chest Hospital and with the 1964 Helsinki declaration and its later amendments or comparable ethical standards.

### Informed consent

Informed consent was obtained from all individual participants included in the study.

### The proposed method

**Figure 1:**
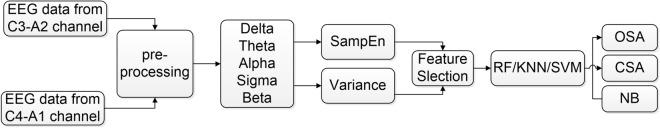
Flow chart of the proposed method, where NB is referred to as normal breathing.

First, EEG signals were deconstructed into five sub-bands, after which the sample entropy and variance of each sub-band are extracted. Next, we applied the nearest neighbor component analysis (NCA) algorithm to feature selection in order to acquire the optimal feature set. Finally, the results were utilized as inputs for the classifiers. The steps for the method are shown in Fig. 1, All procedures of data processing were written in MATLAB R2019b. Figure 1 was obtained with Microsoft Visio 2010.

### Data pre-processing

For data pre-processing, we used an Infinite Impulse Response Butterworth Band pass filter to deconstruct the EEG signals of C3-A2 and C4-A1 into five sub-bands: delta (δ, 0.5–4 Hz), theta (θ,4–8 Hz), alpha (α,8–12 Hz), sigma (σ,12–16 Hz) and beta (β, 16–40 Hz), while filtering out the sensed noise interference outside the frequency band of interest.

### Sub-band feature extraction

#### Sample entropy

The EEG signal is a standard non-linear signal, and the state of the signal may appear randomly on the time scale. Sample entropy, a non-linear statistical method for measurement of the complexity of time series, can effectively handle the randomness of EEG signal^30^. The larger the value of the sample entropy of the signal x, the more complex the sample sequence. The definition of sample entropy is outlined below^31^.

If there is a time series of N sample points {x(i)} = x (1), x (2), …, x(N), where the dimension is *m* (the value of *m* is generally 1 or 2; in this study the value of *m* is 2):

1 $$ {\text{X(i) = [x(i}}{),}{\text{x(i + 1),}}...{\text{,x(i + m - 1)],i = 1,2,}}..{\text{,N - m + 1}} $$

Then, the distance between the vector X(i) and X(j) can be calculated as:

2 $$ {\text{d[X(i),X(j)] = maxk = 0,}}...{\text{m - 1(}}\left| {{\text{x(i}} + {\text{k) - x(j}} + {\text{k)}}} \right|) $$

For a given x(i), we can calculate the number of *j* so that the distance between x(i) and x(j) is lower than or equal to *r* (*r* is a real number, which represents the measure of "similarity"). Generally, *r*=*0.1*~0.25*std(data); herein, we select 0.2 times. For each *i*, we can calculate:

3 $$ C_{{\text{i}}}^{{\text{m}}} (r) = \frac{{num\{ d[X(i),X({\text{j}})] \le {\text{r}\} }}}{{N - {\text{m}} + 1}} $$

*B*^m^(*r*) and *B*^m+1^(*r*) is defined as follows:

4 $$ B^{{\text{m}}} (r) = \frac{1}{N - m}\sum\limits_{i = 1}^{N - m} {C_{i}^{m} } (r) $$

5 $$ B^{{{\text{m}} + 1}} (r) = \frac{1}{N - m}\sum\limits_{i = 1}^{N - m} {C_{i}^{m + 1} } (r) $$

Therefore, the sample entropy is calculated as:

6 $$ {\text{SampEn}}\;({\text{m,r,N) }} = {\text{ - ln}}\left[ {\frac{{{\text{B}}^{{{\text{m}} + {1}}} (r)}}{{B^{m} (r)}}} \right] $$

### Variance

Variance refers to the measure of dispersion of a random variable or a set of data. It is the average of the squared of deviations (the differences between each data point and the mean of the variable). The greater the variance, the greater the deviation from the mean of each sample of signal x, and the greater the degree of dispersion. The formula is for variance is:

7 $$\sigma^{2} = \frac{1}{N}\sum\limits_{{{\text{i}} = 1}}^{N} {(x_{i} - \overline{x} )^{2} }$$

where σ^2^ refers to the variance, *N* refers to the total number of samples, $x_{i}$ refers to the sample value of each epoch, $\overline{x}$ refers to the average.

### Feature selection

Feature selection is a technique that eliminates irrelevant or repetitive features from the original feature set, and chooses a small feature subset, thus decreasing the data dimension and improving the algorithm’s execution speed^32^. The objective of the NCA algorithm, a non-parametric feature selection algorithm for supervised learning, is to learn feature weighing vectors through the use of optimized regular parameters that will allow maximization of the classification accuracy^33^. The algorithm can provide the weight of features, which will allow us to select important features. The steps of NCA algorithm are listed as follows:

1. Divide the data into a training set and a test set
2. Divide the training data into 5 folds (four as the training set and one as the test set) for NCA feature selection
3. Use fivefold cross-validation to tune the regularization parameter λ.
4. For each λ, use the corresponding training set to train the NCA model.
5. Use the trained NCA model to measure the loss of classification of the corresponding test set and obtain the loss value.
6. Repeat steps 2-4.
7. Calculate the average loss of each λ worth 10% cross-validation.
8. Identify the best λ that corresponds to the minimal average loss.
9. Use the test data to determine the weight of each feature using the best λ.
10. Select important features based on feature selection criteria using the relative threshold (T):
  8 $$ T = \tau * \max (w) $$

In which τ is a fixed value of 0.02 and ω is the characteristic weight^34^.

In this algorithm, the best λ is associated with the best classification loss. The average loss of cross-validation is dependent on the choice of λ. When λ gets too large, all the feature weights tend to be 0, which causes redundant or irrelevant feature subsets. Hence, the value of λ should be adjusted to minimize the average loss^34^. Herein, we selected a λ value of 16, with equally spaced points from 0 to 15/N.

### Classifiers

Support vector machine (SVM) and K-nearest neighbor (KNN) are the more common classifiers that are utilized in apnea classification problems, largely due to the fact that the supervised model has learning ability to distinguish binary categories. The random forest (RF) model is an ensemble algorithm, which combines multiple weak classifiers. Then, the final result is voted or averaged, and the result of the overall model has higher accuracy and generalization performance^35^. Therefore, we used RF in this study, as well as SVM and KNN, as classifiers to validate the features obtained above. Due to the imbalance of the data used, the traditional learning algorithm has greater limitations. To solve this problem, we adopted the class weighting method to improve the classification performance by adjusting the weights of different classes to favor minority classes.

### K-nearest neighbor

The KNN classification algorithm, an extension of the nearest neighbor method, is a supervised learning method that belongs to a nonlinear classifier within the classification process^36,37^. KNN classifies by measuring the distance between various feature values. Specifically, if the data and labels of the training set are known, then the characteristics of the test data are compared to the corresponding features within the training data, which can help determine the most similar top k data within the training set. Hence, the test data will belong to the most frequent category among the top k data^38^. The similarity between test data and training data is generally determined by the Euclidean or cosine distance. Herein, we used Euclidean distance.

### Support vector machine

Support vector machine (SVM) is a supervised machine learning algorithm that develops an optimal hyperplane that allows classification of input data^39^. This algorithm was originally designed for solving binary classification problems. The basic principle of SVM is that if the data are points that are distributed on a two-dimensional plane, then they are gathered in different areas as per their classification. The goal of this algorithm is to determine the hyperplane between these categories through training^40^. For multi-class classification problems, a multi-class classifier needs to be developed. Currently, there are two methods for constructing multi-class classifiers, including the direct and the indirect method. In this study, we used the indirect method, which allows division of the multi-class classification problem into multiple two-classification problems. The main idea is to design an SVM for classification into two types of samples. In this study, we carried out SVM classification using LibSVM.

### Random forest

Random forest is an integrated learning algorithm that uses multiple decision trees for prediction. For classification problems, it is the voting of all decision tree prediction results^41^. During training, the training set of each decision tree is constructed using randomly sampling. When training every node of each decision tree, the features that are utilized are also a part of the features that are extracted from the entire feature vector^42^. Through integration of multiple decision trees and training each decision tree with feature components each time, the variance of the model can be efficiently decreased. Theoretically, as the number of decision trees increase, the classification capability of the model also increases. However, at the same time, if the correlation between any two trees increases, then the error rates also increase. Therefore, the optimal number of decision trees also needs to be selected. Herein, the classification of the tree with the best classification effect is 85.

### Performance measures

In order to further assess the performance of each of the three feature sets in the RF classifier, we implemented recall, precision, F_1_-score and kappa coefficient as the evaluation indicators for the classification performance. A confusion matrix contains all the data about the actual label, as well as the predicted label. The Kappa coefficient is utilized for consistency testing, but can also be utilized for measurement of classification accuracy. Its calculation is based on a confusion matrix. The evaluation indicators are defined:

9 $$ recall = \frac{TP}{{TP + FN}} $$

10 $$ precision = \frac{TN}{{TP + FP}} $$

11 $$F_{1}- score = \frac{2 \times precision \times recall}{{precision{\text{ + recall}}}}$$

Here, *TP* refers to true positive (when positive class is classified as positive class), *FP* means false positive (when negative class is classified as positive class), and *FN* refers to false negative (when negative class is classified as positive class).

12 $$k = \frac{p_{o} - p_{e}}{{1 - p_{e}}}$$

In this calculation, p_o_ refers to the sum of the number of samples that are correctly classified within each category, divided by the total number of samples, which provides overall classification accuracy. For p_e_, if we assume that the number of real samples within each category are a_1_, a_2_, ..., a_C_, the predicted number of samples in each category are b_1_, b_2_, ..., b_C_, and the total number of samples is N, then:

13 $$p_{e} = \frac{a_{1} \times b_{1} + a_{2}\times b_{2} + ... + a_{C} \times b_{C}}{{N \times N}}$$

Usually, the calculation of kappa coefficient is between 0 and 1, and the larger the coefficient, the higher the consistency and accuracy.

## Results

Herein, we utilized EEG data from 30 different individuals to detect and classify sleep apnea, as well as analyze the performance of the classifier. The data set contained 3459 epochs, which includes 1229 epochs of OSA events, 812 epochs of CSA events, and 1418 epochs of normal breathing events.

After feature extraction, we obtained 20 feature points for each epoch (Table 1). These 20 feature points constitute the original feature set, and the features of all these epochs form 3459*20-dimensional data.

**Table 1:** The specific meaning of the 20 characteristics.

No	Feature vector		
	feature	Channel	sub-band
1	Variance	C3	Delta
2			Theta
3			Alpha
4			Beta
5			Gamma
6		C4	Delta
7			Theta
8			Alpha
9			Beta
10			Gamma
11	Sample Entropy	C3	Delta
12			Theta
13			Alpha
14			Beta
15			Gamma
16		C4	Delta
17			Theta
18			Alpha
19			Beta
20			Gamma

During the feature selection stage, we first determined the minimum average loss when selecting different training set and test set ratios. Hence, we randomly selected 45%, 40%, 35%, 30%, 25%, and 20% of the original feature set as the test set (Table 2). When 25% of the test set and 75% of the training set were selected, the average loss was found the most minimal.

**Table 2:** The corresponding best loss and best lambda when selecting different test sets.

Test percentage	45%	40%	35%	30%	25%	20%
Best loss	0.1487	0.1594	0.1530	0.1557	0.1455	0.1510
Best lambda	0.0021	0.0009	0.0013	0.0012	0.0011	0.0022

**Figure 2:**
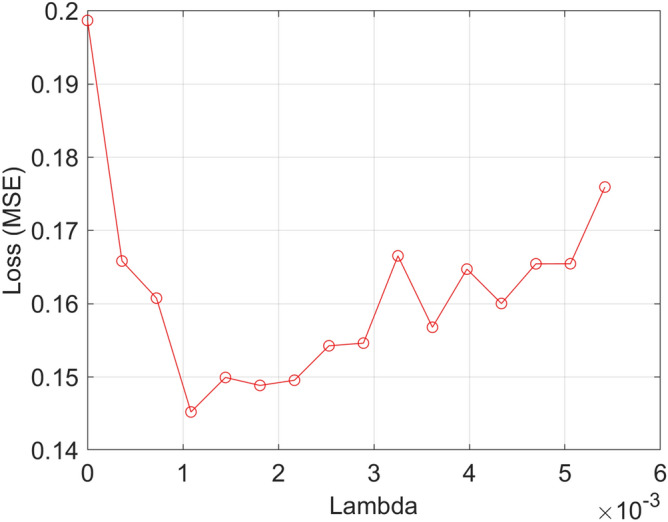
Relationship between average loss and λ value.

The correlation between the average loss and the λ value is shown in Fig. 2. The minimum average loss that corresponded to the 16 lambda values is 0.1445, while the corresponding optimal λ value was 0.0011.

**Figure 3:**
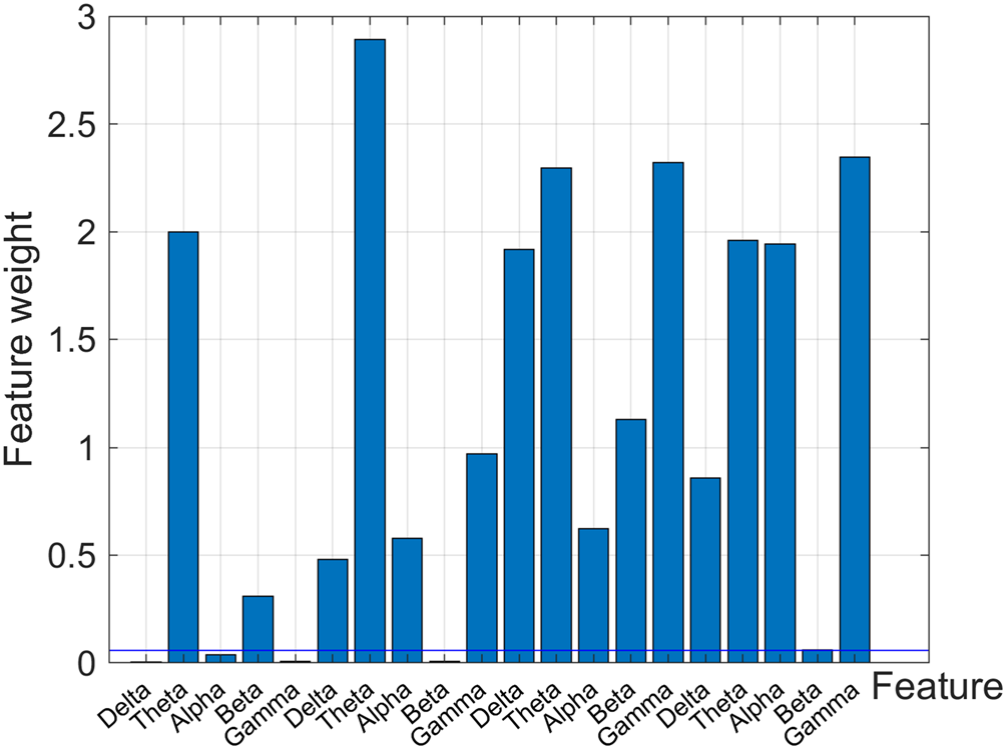
Feature weights after NCA feature selection.

Figure 3 showcases the feature weight of each feature index, and the meaning of the feature that corresponds to each serial number is shown in Table 1. The obtained feature weight that is relative to the threshold T is 0.0567, and 15 important features are screened out according to this value (Table 3).

**Table 3:** The specific meaning of the 15 important features.

No	Feature vector		
	feature	Channel	sub-band
2	Variance	C3	Theta
4			Beta
6		C4	Delta
7			Theta
8			Alpha
10			Gamma
11	Sample Entropy	C3	Delta
12			Theta
13			Alpha
14			Beta
15			Gamma
16		C4	Delta
17			Theta
18			Beta
20			Gamma

**Figure 4:**
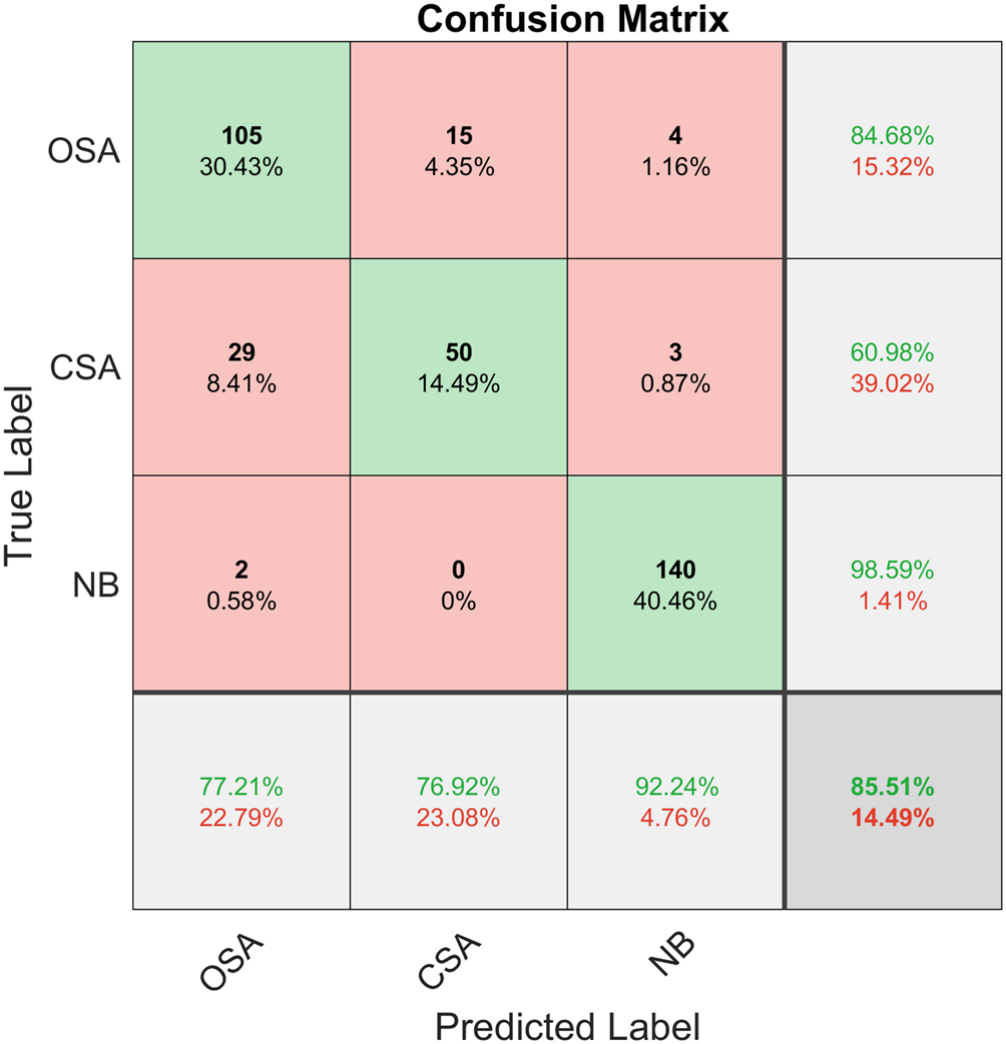
Confusion matrix of KNN classifier classification accuracy.

**Figure 5:**
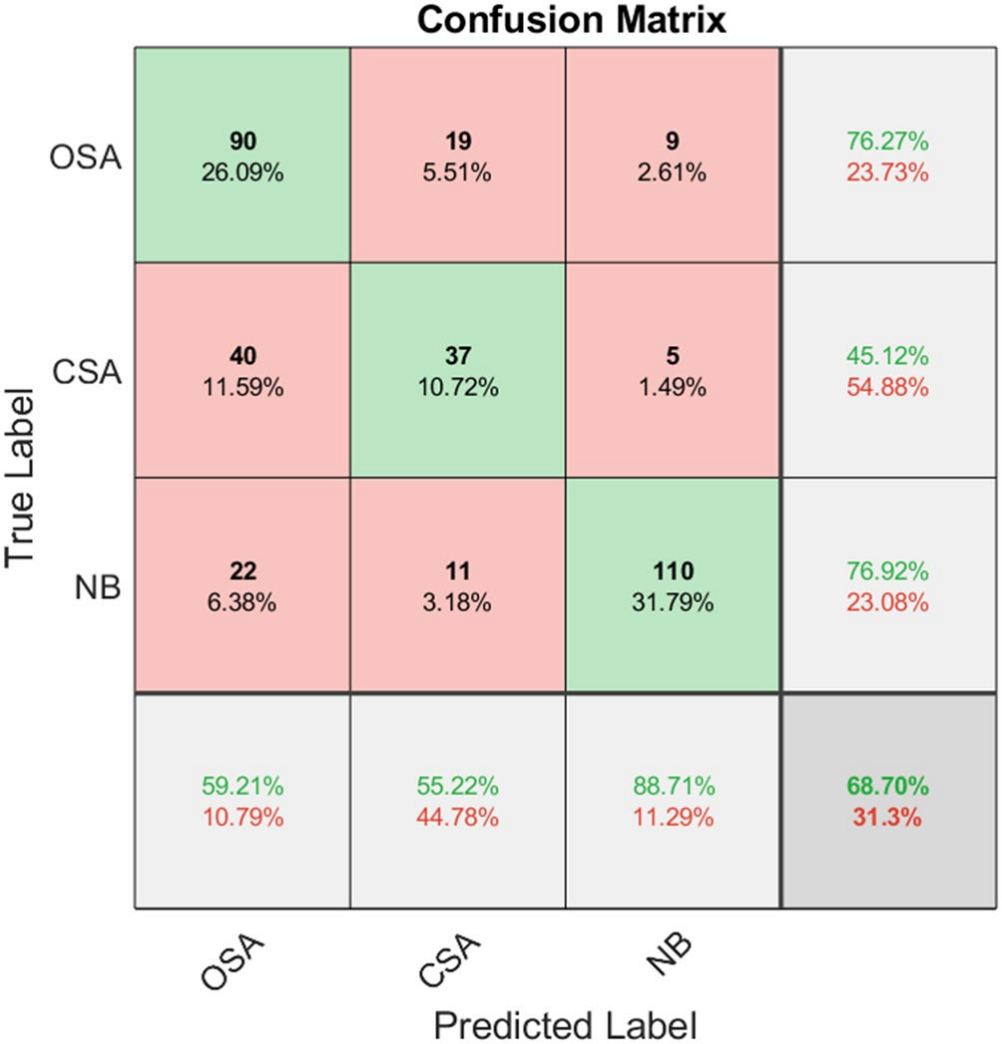
Confusion matrix of SVM classifier classification accuracy.

**Figure 6:**
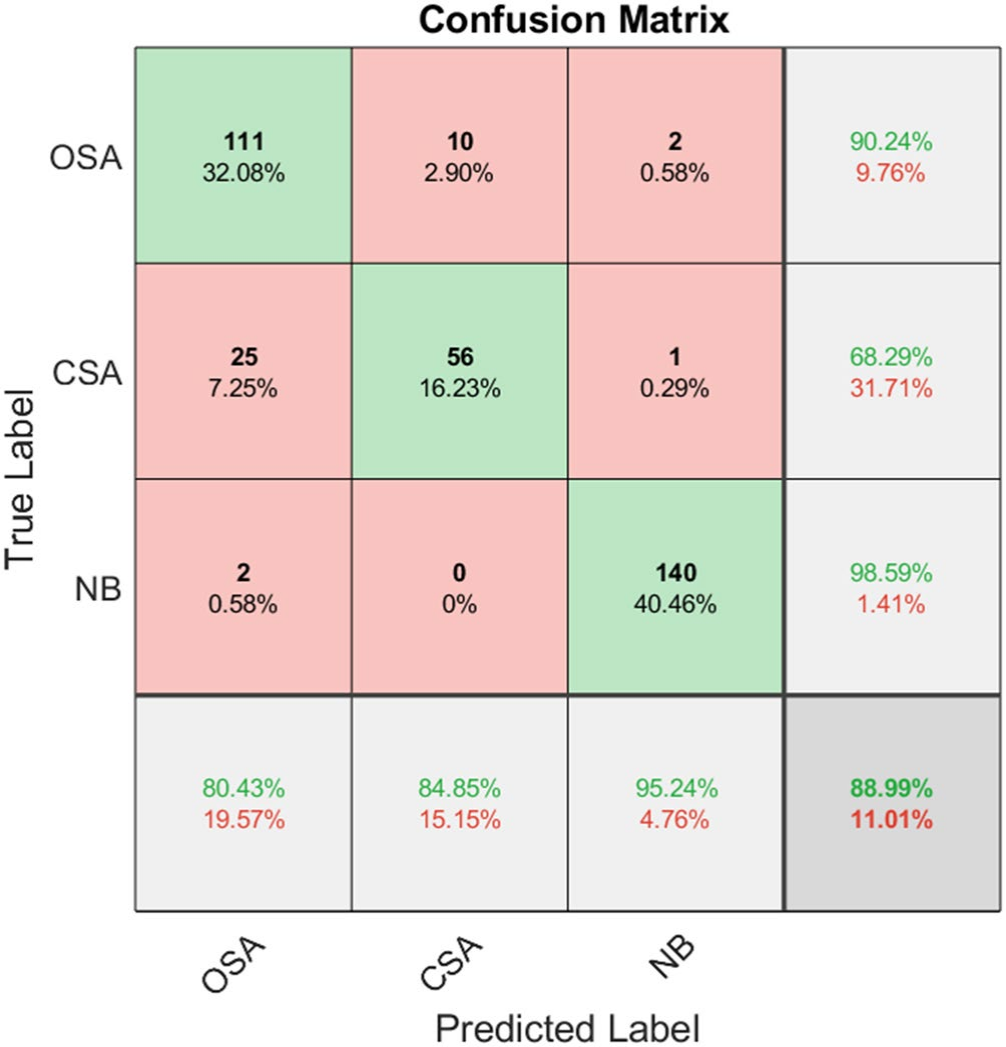
Confusion matrix of RF classifier classification accuracy.

Within the classification stage, we selected the training set as well as the test set by 10-fold cross-validation, allowing us to select the training set and the test set, and input the features into KNN, SVM, and RF for classification. Then, we took the average accuracy of 10-fold cross-validation as the final result. The average confusion matrix of the 10-fold cross-validation of each classifier is given in Figs. 4, 5 and 6.

Table 4 indicates the specific classification results of the three classifiers for obstructive apnea, central apnea and normal breathing. It can be observed from the table that out of three classifiers, RF has the highest classification accuracy, followed by KNN, and SVM has the lowest.

**Table 4:** Classification results of three classifiers.

Classifiers	Average	OSA	CSA	NB
KNN	85.51%	77.21%	76.92%	92.24%
SVM	68.70%	59.21%	55.22%	88.71%
RF	88.99%	80.43%	84.85%	95.24%

Table 5 demonstrates the classification performance of the three classifiers. RF was shown to have the highest classification accuracy and the best classification effect. It can be seen that the random forest classifier has better recall, accuracy, F1-score and kappa coefficient than the other two classifiers, and has the best classification performance.

**Table 5:** Classification performance of three classifiers, including accuracy, recall, F1-score and Kappa coefficient.

Classifiers	Recall	Precision	F1-score	kappa
KNN	0.81	0.82	0.82	0.83
SVM	0.67	0.68	0.67	0.54
RF	0.86	0.89	0.87	0.85

## Discussion

In this study, we propose a method that will allow classification of sleep apnea by applying classifiers to EEG signals. In order to obtain a high performing classifier, it is necessary to eliminate the non-contributing elements within the original feature data set. Therefore, we utilized NCA to carry out feature selection in the original data set to acquire a data set that contained 13 features. Finally, three classifiers are utilized to detect and classify sleep apnea. The results of this feature selection indicate the presence of six features in the important feature subset from the left hemisphere EEG signal (C3-A2), and seven features from the right hemisphere (C4-A1). This indicates that patients with sleep apnea have different changes in the EEG of the central scalp area of the left (C3-A2) and right (C4-A1) during sleep, specifically, the right cerebral hemisphere is more active than the left hemisphere during sleep. Additionally, it can be concluded from Fig. 3 that the feature weight that is taken from the sub-band theta is the larger of all the five sub-bands, and the feature weight of the low-frequency sub-band is bigger than that of the high-frequency sub-band. This illustrates the importance of low-frequency EEG components that allow detection of sleep apnea, which is consistent with literature^43^.

There are also the studies that devoted to automatic classification of OSA events, CSA events and normal breathing events based on EEG signals. Monika et al. used discrete wavelet transform and Hilbert transform in order to extract statistical characteristics, including instantaneous amplitude, instantaneous frequency, and weighted instantaneous frequency from EEG epochs. In order to select features, they utilized variance analysis and multiple regression analysis. Then, using a feed-forward neural network, they reported that the average accuracy was 77.27%, while the accuracy for normal breathing, OSA, and CSA were 71.21%, 86.36% and 74.24%, respectively^14^. They also used the same feature extraction and selection method to analyze the EEG signals of C3-A2 and C4-A1 channels, and concluded that combining the two EEG channels together is more effective at automatic detection and discrimination of sleep apnea^43^. In comparison to these studies, the proposed automatic classification method for sleep apnea has high accuracy.

## Conclusion

In this study, a classification algorithm that was based on EEG sub-band signal feature extraction is proposed to help classify sleep apnea events. The EEG signals used in this study came from a database of night polysomnography among patients treated at the Tianjin Chest Hospital. The results of this study show that the average accuracy of random forest classifier classification can reach 88% after feature selection using the NCA feature selection algorithm, while the accuracy, the recall rate, and the F1-score was 0.86, 0.89, and 0.87, respectively. Hence, this method can automatically detect the occurrence of sleep apnea events, determine the type of apnea event, and, to some extent, replace sleep experts to complete the diagnosis of respiratory events, thereby saving medical resources and time. The classification of apnea events based on EEG signals is only a preliminary study for now. In the future research, we will further improve accuracy on this basis, and work on classifying sleep apnea types and severity to develop a set of methods determining sleep apnea and severity based on EEG.

## Data Availability

The data used and analyzed during the current study are available from the corresponding author on reasonable request.

## References

[1] Flemons WW (1999). Sleep-related breathing disorders in adults: Recommendations for syndrome definition and measurement techniques in clinical research. Sleep.

[2] Maurer JT (2008). Early diagnosis of sleep related breathing disorders. GMS Curr. Top. Otorhinolaryngol. Head Neck Surg..

[3] Cao, M. T., Guilleminault, C., Kushida, C. A. J. P. & Medicine, P. O. S. Clinical features and evaluation of obstructive sleep apnea and upper airway resistance syndrome. In *Principles & Practice of Sleep Medicine*. Chapter 105, 1206–1218 (2011).

[4] Peppard PE, Young T, Palta M, Skatrud J (2000). Prospective study of the association between sleep-disordered breathing and hypertension. N. Engl. J. Med..

[5] Thorpy, M. & Goswami, M. In *Handbook of Sleep Disorders* (ed. Kushida, C.A) 351–364 (Marcel Dekker, New York, 1990).

[6] Flemons WW (1999). Sleep related breathing disorders in adults: Recommendations for syndrome definition and measurement techniques in clinical research. Sleep.

[7] Penzel T (2002). Systematic comparison of different algorithms for apnea detection based on electrocardiogram recordings. Med. Biol. Eng. Comput..

[8] Tagluk ME, Sezgin N (2010). Classification of sleep apnea through sub-band energy of abdominal effort signal using Wavelets + Neural Networks. J. Med. Syst..

[9] Schlueter T, Conrad S (2012). An approach for automatic sleep stage scoring and apnea-hypopnea detection. Front. Comput. Sci. China.

[10] Schultz SK (2001). Principles of neural science. Am. J. Psychiat..

[11] See, A. R. & Liang, C. K. A study on sleep EEG Using sample entropy and power spectrum analysis. In *Defense Science Research Conference & Expo (DSR)*, Vol. 3, 1–4 (2011).

[12] Uçar MK, Bozkurt MR, Bilgin C, Polat K (2016). Automatic detection of respiratory arrests in OSA patients using PPG and machine learning techniques. Neural. Comput. Appl..

[13] Uddin MB, Chow CM, Su SW (2018). Classification methods to detect sleep apnea in adults based on respiratory and oximetry signals: A systematic review. Physiol. Meas..

[14] Prucnal, M. A. & Polak, A. G. Analysis of features extracted from EEG epochs by discrete wavelet decomposition and Hilbert transform for sleep apnea detection. In *Annual International**Conference of the IEEE Engineering in Medicine and Biology Society* 287–290 (2018). 10.1109/EMBC.2018.8512201 30440394

[15] Acir N, Guzelis C (2004). Automatic recognition of sleep spindles in EEG by using artificial neural networks. Expert Syst. Appl..

[16] Duman F, Erdamar A, Eroğul O, Telatar Z, Yetkin S (2009). Efficient sleep spindle detection algorithm with decision tree. Expert Syst. Appl..

[17] Aydin S (2011). Computer based synchronization analysis on sleep EEG in Insomnia. J. Med. Syst..

[18] Saha, S., Bhattacharjee, A., Ansary, M. A. A. & Fattah, S. A. An Approach for Automatic Sleep Apnea Detection Based on Entropy of Multi-Band EEG Signal. In *2016 IEEE Region 10 Conference (TENCON),* 420–423 (2016).

[19] Black JE, Guilleminault C, Colrain IM, Carrillo O (2000). Upper airway resistance syndrome-central electroencephalograp-hic power and changes in breathing effort. Am. J. Respir. Crit. Care Med..

[20] Sugi T, Kawana F, Nakamura M (2009). Automatic EEG arousal detection for sleep apnea syndrome. Biomed. Signal Process. Control..

[21] Tagluk ME, Sezgin N (2011). A new approach for estimation of obstructive sleep apnea syndrome. Expert Syst. Appl..

[22] Almuhammadi W.S., Aboalayon K.A., Faezipour M.: Efficient obstructive sleep apnea classification based on EEG signals. In *IEEE Long Island Systems, Applications and Technology Conf (LISAT)*, May, 1–6 (2015).

[23] Zhou J, Wu XM, Zeng WJ (2015). Automatic detection of sleep apnea based on EEG detrended fluctuation analysis and support vector machine. J. Clin. Monitor. Comput..

[24] Bhattacharjee A (2019). Sleep Apnea detection based on rician modeling of feature variation in multiband EEG signal. IEEE. J. Biomed. Health. Inform..

[25] Saha S, Bhattacharjee A, Fattah SA (2019). Automatic detection of sleep apnea events based on inter-band energy ratio obtained from multi-band EEG signal. Healthc. Technol. Lett..

[26] Ahmed, F., Paromita, P., Bhattacharjee, A., Saha, S. & Fattah, S. A. Detection of sleep apnea using sub-frame based temporal variation of energy in beta band in EEG. In *2016 IEEE International WIE Conference on Electrical and Computer Engineering (WIECON-ECE)*. 2016, 258–261 (2016).

[27] Shahnaz, C., Minhaz, A. T. & Ahamed, S. T. Sub-frame based Apnea detection exploiting delta band power ratio extracted from EEG signals. In *TENCON 2016–2016 IEEE Region 10 Conference.* 2016, 190–193 (2016).

[28] Taran S, Bajaj V, Sharma D (2017). Robust Hermite decomposition algorithm for classification of sleep apnea EEG signals. Electron. Lett..

[29] Taran S, Bajaj V (2020). Sleep apnea detection using artificial bee colony optimize hermite bfunctions for EEG signals. IEEE Trans. Instrum. Meas..

[30] Richman JS, Moorman JR (2000). Physiological time-series analysis using approximate entropy and sample entropy. Am. J. Physiol. Heart Circul. Physiol..

[31] Goldberger, J., Roweis, S. T., Hinton, G. E. & Salakhutdinov, R. Neighborhood components analysis. In *Advances in Neural Information Processing Systems (ANIPS)*, Vol. 17 (2004).

[32] Liu H, Motoda H (1998). Feature Selection for Knowledge Discovery and Data Mining.

[33] Raghu S, Sriraam N (2018). Classification of focal and non-focal EEG signals using neighborhood component analysis and machine learning algorithms. Expert Syst. Appl..

[34] Yang W, Wang K, Zuo WJJOC (2012). Neighborhood component feature selection for high-dimensional data. J. Comput..

[35] Avci C, Akbas A (2015). Sleep apnea classification based on respiration signals by using ensemble methods. Biomed. Mater. Eng..

[36] Vimala V, Ramar K, Ettappan M (2019). An intelligent sleep apnea classification system based on EEG signals. J. Med. Syst..

[37] Franco-Lopez H, Ek AR, Bauer ME (2001). Estimation and mapping of forest stand density, volume, and cover type using the k-nearest neighbor method. Remote Sens. Environ..

[38] Guo GD, Wang H, Bell D, Bi YX, Greer K, Meersman R, Tari Z, Schmidt DC (2003). KNN model-based approach in classification. On the Move to Meaningful Internet Systems.

[39] Abedi Z, Naghavi N, Rezaeitalab F (2017). Detection and classification of sleep apnea using genetic algorithms and SVM-based classification of thoracic respiratory effort and oximetric signal features. Comput. Intell..

[40] Fu K, Qu JF, Chai Y, Dong Y (2014). Classification of seizure based on the time-frequency image of EEG signals using HHT and SVM. Biomed. Signal Process. Control.

[41] Ren, Q., Cheng, H. & Han, H., Research on Machine Learning Framework Based on Random Forest Algorithm in Advances in Materials, Machinery, Electronics I (ed. L. Liu, C. Yang, J. Ke) Vol. 1820 (Amer Inst Physics, 2017).

[42] Biau GJJOMLR (2010). Analysis of a sodel. J. Mach. Learn. Res..

[43] Prucnal MA, Polak AG (2019). Comparison of information on sleep apnoea contained in two symmetric EEG recordings. Metrol. Meas. Syst..

